# Treatment and Staging Intensification Strategies Associated with Radical Prostatectomy for High-Risk Prostate Cancer: Efficacy Evaluation and Exploration of Novel Approaches

**DOI:** 10.3390/cancers16132465

**Published:** 2024-07-05

**Authors:** Giuseppe Reitano, Tommaso Ceccato, Simone Botti, Martina Bruniera, Salvatore Carrozza, Eleonora Bovolenta, Gianmarco Randazzo, Davide Minardi, Lorenzo Ruggera, Mario Gardi, Giacomo Novara, Fabrizio Dal Moro, Fabio Zattoni

**Affiliations:** 1 Department of Urology, Azienda Ospedale-Università Padova, 35122 Padova, Italy; reitano.giuseppe@mayo.edu (G.R.); tommaso.ceccato@aopd.veneto.it (T.C.); simone.botti@aopd.veneto.it (S.B.); martina.bruniera@aopd.veneto.it (M.B.); salvatore.carrozza@studenti.unipd.it (S.C.); eleonora.bovolenta@aopd.veneto.it (E.B.); gianmarco.randazzo@aopd.veneto.it (G.R.); davide.minardi@aopd.veneto.it (D.M.); lorenzo.ruggera@aopd.veneto.it (L.R.); mario.gardi@aopd.veneto.it (M.G.); giacomo.novara@unipd.it (G.N.); fabrizio.dalmoro@unipd.it (F.D.M.); 2 Department of Urology, Mayo Clinic, Rochester, MN 55905, USA; 3 Department of Medicine (DIMED), University of Padua, 35128 Padova, Italy

**Keywords:** high-risk prostate cancer, neoadjuvant therapy, adjuvant therapy, PSMA-PET, prostatectomy, lymphadenectomy

## Abstract

**Simple Summary:**

High-risk prostate cancer is an aggressive disease, and its treatment can be complex and require the involvement of several specialists. Advances in imaging and therapies in this field can improve survival and help physicians choose the best personalized approach that maintains quality of life. This article summarizes the most recent publications on this condition and its treatments, aiming to provide an updated guide for managing patients with prostate cancer who experience a higher risk of progression and death.

**Abstract:**

The management of high-risk prostate cancer (PCa) presents a significant clinical challenge, often necessitating treatment intensification due to the potential presence of micrometastases. While radical prostatectomy (RP) constitutes one of the primary treatment modalities, the integration of neoadjuvant and adjuvant therapies suggests a paradigm shift towards more aggressive treatment strategies, also guided by new imaging modalities like positron emission tomography using prostate-specific membrane antigen (PSMA-PET). Despite the benefits, treatment intensification raises concerns regarding increased side effects. This review synthesizes the latest evidence on perioperative treatment intensification and de-escalation for high-risk localized and locally advanced PCa patients eligible for surgery. Through a non-systematic literature review conducted via PubMed, Scopus, Web of Science, and ClinicalTrials.gov, we explored various dimensions of perioperative treatments, including neoadjuvant systemic therapies, adjuvant therapies, and the role of novel diagnostic technologies. Emerging evidence provides more support for neoadjuvant systemic therapies. Preliminary results from studies suggest the potential for treatments traditionally reserved for metastatic PCa to show apparent benefit in a non-metastatic setting. The role of adjuvant treatments remains debated, particularly the use of androgen deprivation therapy (ADT) and adjuvant radiotherapy in patients at higher risk of biochemical recurrence. The potential role of radio-guided PSMA lymph node dissection emerges as a cutting-edge approach, offering a targeted method for eradicating disease with greater precision. Innovations such as artificial intelligence and machine learning are potential game-changers, offering new avenues for personalized treatment and improved prognostication. The intensification of surgical treatment in high-risk PCa patients is a dynamic and evolving field, underscored by the integration of traditional and novel therapeutic approaches. As evidence continues to emerge, these strategies will refine patient selection, enhance treatment efficacy, and mitigate the risk of progression, although with an attentive consideration of the associated side effects.

## 1. Introduction

High-risk (HR) localized prostate cancer (PCa) accounts for less than a quarter of all PCa [1]. HR PCa is more likely to have micrometastatic disease that can be missed both by conventional and molecular imaging [2]. HR PCa is related to worse oncological outcomes and has an increased mortality rate [3]. The management of these patients is complex and often requires a multimodal approach that combines surgery, radiotherapy, and systemic treatment [4]. The ongoing research is focused on trying to find a tailored approach for the treatment of HR PCa based on different biochemical, pathological, and clinical features. Physicians must combine the best treatment options to maximize oncological radicality while preserving the quality of life of the patients, avoiding unnecessary treatment, and minimizing related side effects [5]. Following these purposes, it is important to understand when it is appropriate to intensify the treatments and when it is preferable to intensify the staging and diagnostic approach to limit the aggressiveness of certain surgical maneuvers and non-surgical procedures. Traditionally, prostate cancer management relied on standard imaging techniques like computed tomography (CT) and bone scintigraphy [6]. However, these methods often miss metastatic lesions when PSA levels are low, hindering early intervention [7]. Recently, PSMA-PET has changed the staging approach [8]. This allows for a more targeted therapeutic strategy, potentially adjusting the initially planned treatment regimen [9,10]. Finally, artificial intelligence (AI) may be a resource in the management of HR PCa and its use and application are growing in many fields [11]. This review aims to provide an updated overview of the most recent findings regarding the current management of HR PCa candidates to a definitive surgical treatment, with a separate focus on the role of PSMA-PET as a tool to evaluate the results of the therapies in this multidisciplinary setting.

## 2. Materials and Methods

We performed a non-systematic search on PubMed, Scopus, Web of Science, and ClinicalTrials.gov to identify the most relevant studies published in English in the last 5 years. The keywords used were “prostate cancer”, “radical prostatectomy”, “neoadjuvant”, “adjuvant therapy”, “adjuvant radiotherapy”, “adjuvant androgen deprivation therapy”, “adjuvant chemotherapy”, “adjuvant complete androgen blockade” “PSMA-PET”, “artificial intelligence”, “guided lymphadenectomy”, and “PSMA-PET guided surgery”. Boolean operators were used as appropriate to enhance the search. Medical Subject Heading phrases (MeSH) were also used to refine the screening.

## 3. PSMA-PET for Intensification or De-Intensification of Surgical Therapy

PSMA-PET has higher sensitivity and specificity in the detection of metastases when compared to conventional CT scans or bone scans; this may help identify patients with systemic disease who are likely to benefit from systemic treatment rather than local therapies after the initial diagnosis [9,10]. PSMA-PET is becoming a key tool in the planning and optimization of salvage therapies for patients with PSA persistence, as well as for those who have already received previous salvage treatments [12]. Up until now, new-generation imaging has not been capable of detecting micrometastases [13]. However, PSMA-PET might be a resource for the selection of patients who may be candidates for neoadjuvant therapy before radical prostatectomy and it may be useful in the post-treatment evaluation to re-stage the patients and try to make considerations on the effect of systemic preoperative treatment [14].

PSMA-PET/CT has emerged as a potential predictor of favorable pathological response to neoadjuvant treatments before RP. Standardized uptake value (SUVmax) after treatment may be used in the future for predicting the pathological response in HR patients treated with ADT and abiraterone acetate or ADT and docetaxel before surgery. It seems that patients with favorable [68Ga]PSMA-PET/CT response after neoadjuvant treatment (SUVmax ≤ 8.5) or favorable pathologic response had significantly lower rates of 3-year biochemical progression [9].

Moreover, in HR non-metastatic Pca, [68Ga]Ga-PSMA-11 PET/CT shows promise as a superior predictor of favorable pathological response compared to multiparametric magnetic resonance imaging (mpMRI) and nadir PSA. In fact, in a recent prospective study, EAU/EANM recommended criteria and PERCIST 1.0 criteria performed equally well in identifying pathological responders when [68Ga]Ga-PSMA-11 PET/CT was used as a therapeutic response assessment tool before RP in HR non-metastatic patients undergoing neoadjuvant therapy [15].

Taken together, despite the small number of patients involved, these studies offer an interesting use of PSMA-PET/CT among patients receiving neoadjuvant treatments prior to surgery.

PSMA may help the surgeon in the next years in the choice of a surgical approach; indeed, lymph node dissection (LND) may be limited to those suspected lymph nodes identified after the administration of the tracer [16]. Preliminary studies showed high intraoperative concordance between preoperative imaging results and the tracer used during surgery [16]. The identification of patients who may be candidates to a de-intensified LND is a way to reduce morbidity and length of stay, improving patients care. PSMA-PET outperformed conventional validated nomograms (Briganti 2019, Memorial Sloan Kettering Cancer Center) in the prediction of lymph node involvement and, as soon as more evidence emerges, may offer an opportunity to spare the lymphadenectomy in 80–90% of patients with a nomogram result that is higher than the predefined cut-off for performing LND [17]. Higher intraprostatic SUVmax values after PSMA-PET seem to be associated with a higher likelihood of lymph node involvement and with distant metastases, representing a potential indicator of aggressive diseases which may benefit from a more intense treatment [14]. Anyway, the retrospective nature of this finding should be carefully evaluated and further proved in well-structured studies since this may be an additional option for a personalized therapy.

PSMA-PET can be useful to intensify postoperative treatment, introduce systemic therapies, or target the treatment based on the imaging outcome. This may improve cancer control, enhance quality of life, and reduce toxicity [18]. The last update of the AUA guidelines indeed recommends that PSMA-PET may be used in case of biochemical recurrence (BCR) after local therapy as an alternative to other imaging techniques or after negative conventional staging. The positive rate associated with PSMA-PET is roughly 40% when the PSA is less than 0.2 ng/mL [19].

## 4. Neoadjuvant Treatment before Radical Prostatectomy

**Figure 1:**
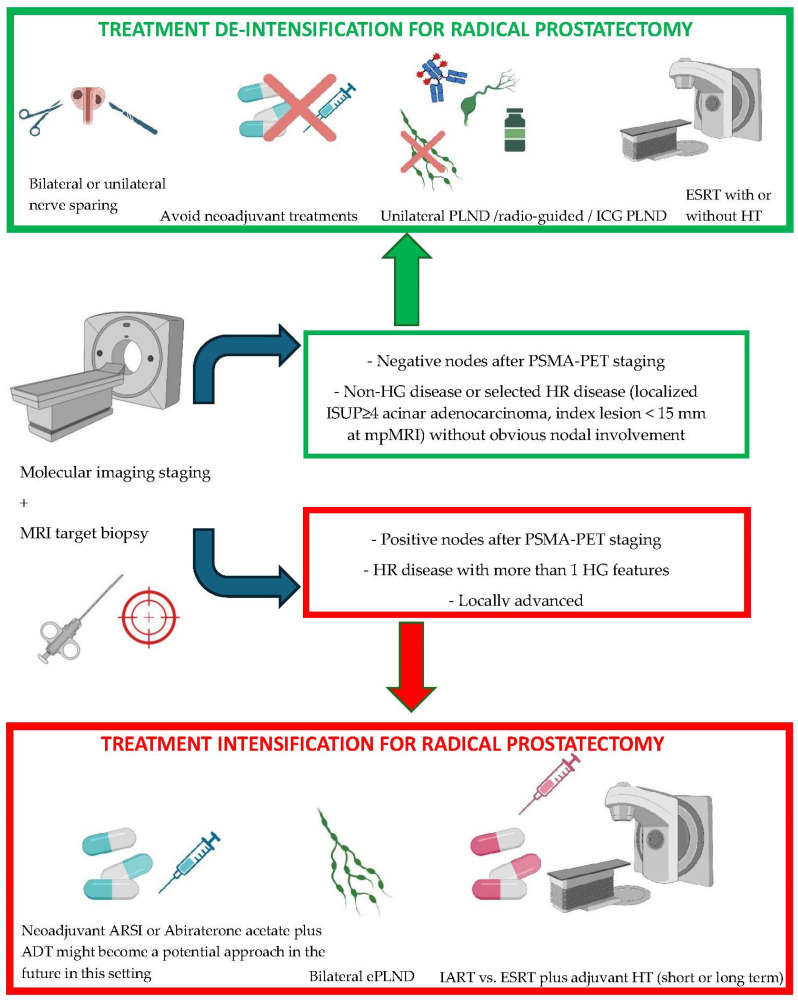
Intensification and de-intensification of perioperative and intraoperative approaches. Patients with non-HR PCa and those with more localized HR PCa may represent the target population for a de-intensified treatment approach. For instance, extended lymphadenectomies could potentially be avoided, while unilateral and bilateral nerve-sparing approaches may be safer options. Patients with locally advanced prostate cancer with a higher number of HR features might benefit from neoadjuvant systemic therapies in the future. Anyway, this subset of patients should be careful managed within a multidisciplinary approach aimed to maximize the survival benefits. (PLND = pelvic lymph node dissection; ESRT = early salvage radiotherapy; HR = high risk; HG = high grade; ISUP = International Society of Urological Pathology; mpMRI = multiparametric magnetic resonance imaging; ARSI = androgen receptor signaling inhibitor; ADT = androgen deprivation therapy; ePLND = extended pelvic lymph node dissection; IART = image-guided adjuvant radiotherapy; HT = hormone therapy). Created with https://BioRender.com.

There are several neoadjuvant pre-surgical strategies that have been explored in the past years. The results published on this topic are still too immature to provide a definitive indication and a target population who might benefit the most from this treatment. Improvement from the pathological standpoint and potential benefit from an oncological perspective must be balanced with side effects and concerns on the delay of the surgical treatment for HR PCa patients. Findings related to neoadjuvant therapy are summarized in Table 1 and Figure 1.

### 4.1. Androgen Deprivation Therapy before Radical Prostatectomy

Androgen deprivation therapy (ADT) is an established treatment for metastatic prostate cancer and non-metastatic patients in several situations; however, the use of neoadjuvant androgen deprivation therapy (NADT) prior to RP is not recommended by EAU and ASCO guidelines [48,57]. Indeed, it is known that NADT alone cannot completely interfere with the androgen biosynthesis in the adrenals and in the tumor itself [58].

There are no recent clinical trials focused on the use of ADT alone or in combination with first-generation antiandrogens before RP. A meta-analysis summarized the findings of the last randomized clinical trials (RCTs) on NADT before RP or radiotherapy (RT). Overall, this treatment seems to improve the positive margin rate, reducing the volume of the disease, and it also has a positive impact on the lymph node rate among HR Pca. Looking at the overall and disease-free survival, amidst the four studies which investigated at least one of these two outcomes in patients undergoing radical prostatectomy, no one was able to detect an improvement in the two oncological endpoints [20].

A review of the most recent publications has identified a few retrospective studies examining the use of NADT [21,22]. It is not always easy to evaluate the role of the neoadjuvant treatments, since many patients with HR Pca who receive preoperative therapies then need adjuvant or salvage treatments as well, and this factor can modify many oncological outcomes, making the role of the preoperative therapies less clear [32]. It must be noted that the evidence available suggests that neoadjuvant therapy may improve many pathological outcomes, including Gleason score (GS) and cancer weight, reducing also the operative time, blood loss, and lowering the rate of positive surgical margins among HR Pca when compared to surgery alone [20,21,22]. Nevertheless, the role of NADT in the improvement of survival outcomes is still unclear, indeed there are no benefits on short or long-term oncological outcomes [20,22]. For conventional NADT the main limitation is that the most recent evidence predominantly originates from retrospective studies. Nonetheless, their findings are consistent with previous, more rigorous works [20]. Finally, the new clinical trials are mainly focused on the use of new molecules (second-generation antiandrogen or abiraterone acetate) in combination with the traditional ADT rather than on the ADT alone [25,26,27,28].

### 4.2. Abiraterone Acetate before Radical Prostatectomy

Abiraterone works by blocking an enzyme CYP17A1, which is involved in the biosynthesis of androgens.

A pooled analysis of two phase II RCTs (NCT04356430 and NCT04869371) encompassed 150 very-high-risk Pca patients followed for more than 3 years (median follow-up of 42 months). The trials compared the efficacy and safety of ADT plus docetaxel or ADT plus abiraterone versus ADT alone prior to RP. The analysis has shown a significant difference in terms of pathological complete response (absence of carcinoma in the prostatectomy specimen) and minimal residual disease rates (defined as residual tumor < 5 mm) [24].

Intriguing results came from a phase II RCT that compared abiraterone and LHRH-analogous vs. LHRH-analogous alone in HR Pca. This study found that abiraterone might not improve conventional pathological outcomes when compared to ADT alone; however, the reduction in tumor volume appeared higher and no patient experienced BCR at a median follow-up of 4 years in the abiraterone arm [23]. Despite the small amount of evidence, these outcomes show that the use of abiraterone acetate in combination with ADT may reduce the volume of the disease in HR patients and could possibly provide a benefit also in terms of BCR, although further research is needed to confirm these effects.

### 4.3. Androgen Receptor Signaling Inhibitors before Radical Prostatectomy

Androgen receptor signaling inhibitors (ARSIs) are drugs extensively used in metastatic and locally advanced settings [59]. Several phase II trials evaluated the role of these treatments as neoadjuvant medications prior to RP. We identified the most recent and relevant phase II trials focused on pathological outcomes, the NEAR trial (apalutamide) [28], ARNEO trial (apalutamide) [26], the SUGAR trial (darolutamide) [25], and the NCT02430480 trial (enzalutamide) [27]. The NEAR trial aimed to evaluate the role of a 12-week course of neoadjuvant apalutamide alone at a dose of 240 mg without a comparison group; the primary endpoint (pathological complete response) was not reached. However, the addition of a second hormonal therapy seemed to increase the likelihood of pathological response [28]. In fact, in the ARNEO trial, investigators found that the combination of degarelix plus apalutamide (240 mg) for 12 weeks in the preoperative setting in 89 high-risk patients gave a clinically relevant pathological response, measured as minimal residual diseases (MRDs, residual cancer burden ≤ 0.25 cm^2^). In this trial, 38% of the patients who received the combination therapy met the primary endpoint versus 9% in those who received degarelix alone. While a downstaging in Pca with clinically node-positive (cN+) disease was observed, a pathological downstaging was seen only in half of the patients [26]. The SUGAR trial is the first trial to investigate the use of perioperative darolutamide at a dose of 600 mg administered twice daily for 9 months vs. the standard of care (SOC), radical prostatectomy. This trial will be the first randomized trial comparing a single neoadjuvant ARSI to the SOC. The results are eagerly awaited [25]. The NCT02430480 trial involved 36 patients with high-risk disease. They were treated with enzalutamide plus ADT (LHRH-agonist or antagonist based on the clinician’s preference) for 6 months. After RP, a complete response was found in only two patients and 13 Pcas had an MRD < 0.05 cc. Although the primary endpoint was related to the magnetic resonance results, the pathological outcomes seem to be consistent with other studies on ARSIs [27]. To the best of our knowledge, the most recent phase II RCT [60,61] focused on ARSI combination therapy (ARSIs plus abiraterone acetate plus ADT) for maximal androgen blockade did not show any advantage when compared to a single ARSI plus ADT. Neoadjuvant ARSIs might be explored in the future as an interesting option also for patients with unresectable tumors, making them surgical candidates and extending the indication for surgery [31]. However, current evidence is lacking. Thus, ARSI neoadjuvant treatment might become a promising strategy that may provide better results in terms of surrogate oncological outcomes (MRD, PR, PCR) compared to standard ADT and does not appear to increase perioperative complications when compared to ADT alone, though the surrogate outcomes are not strongly associated with the main survival outcomes [25,26,27,28]. Unfortunately, the small samples of patients involved limit the opportunity to draw conclusions about the use of ARSIs before RP. The papers on this topic cannot help in selecting the best subgroup of HR patients for a preoperative systemic therapy. Overall, ARSIs may reduce the tumor burden, having a positive impact on the pathological adverse features, so they might be useful, especially among patients with a higher tumor burden or nodal disease. Having fewer positive margins or less locally advanced disease could reduce the BCR rates and prolong the BCR-free survival [29,30].

### 4.4. Chemotherapy before Radical Prostatectomy

Among the literature available, there are some clinical trials focused on chemotherapy as a neoadjuvant treatment before surgery. In 2020, the results of a relevant clinical trial were published. The study involved 738 patients who underwent either neoadjuvant treatment with docetaxel plus surgery or surgery alone. The primary endpoint of this study was not met, since they found no difference in the 3-year biochemical progression-free survival (BPFS) between the two treatment arms, but they showed that patients treated with neoadjuvant chemo-hormonal therapy had less local progression and an improvement in metastasis-free survival (MFS), overall survival (OS), and event-free survival, suggesting that men who received RP without systemic therapies were more prone to receive additional subsequent treatments. It must be considered that all the patients received neoadjuvant ADT and some patients also received adjuvant or salvage radiotherapy and/or adjuvant ADT as a multimodal treatment; these factors affected the power of the primary endpoint for the neoadjuvant therapy alone [32]. In 2019, Narita et al. investigated the role of neoadjuvant docetaxel plus estramustine in HR-localized Pca undergoing RP. They highlighted that this neoadjuvant chemotherapy regimen was able to prolong biochemical recurrence-free survival but at the price of more frequent surgical complications. It must be noted that the lack of randomization could negatively impact their results [34].

A recent phase II RCT tested the role of cabazitaxel plus abiraterone and LHRH agonist in a neoadjuvant setting before RP but found no clinical benefit in terms of pathological complete response and MRD when these treatments were provided to HR Pca patients compared to abiraterone and ADT without chemotherapy [33].

Neoadjuvant chemotherapy is generally investigated along with hormonal treatment and the evidence supporting its use is very limited; indeed, the impact on survival and metastasis probability over a long period is modest and must be balanced with the side effects related to the systemic treatment, in particular grade 3 and 4 adverse events (neutropenia, hyperglycemia, febrile neutropenia) and an apparent greater tendency to postoperative bleeding [33,34]. At present, the studies on neoadjuvant chemotherapy remain too heterogeneous to be compared and to be used as a guide in clinical practice. Moreover, systemic ARSIs or abiraterone acetate prior to surgery might result in better pathological outcomes and fewer adverse events compared to chemotherapy in localized HR Pca [33]. Eventually, chemotherapy should not be used as a neoadjuvant therapy prior to RP in patients with localized HR Pca outside of a clinical trial.

### 4.5. [177Lu]Lu-PSMA-617 before Radical Prostatectomy

[177Lu]Lu-PSMA-617 is a radioligand therapy based on the target delivery of radiation to sites of prostate cancer. It is mainly used in patients with metastatic castration-resistant prostate cancer (mCRPC). A recent single-arm phase I/II trial involved patients with non-metastatic high-risk prostate cancer and divided them into two groups to receive a single cycle or two cycles of [177Lu]Lu-PSMA-617 before surgery. They found that this highly targeted radiotherapy can produce a significant reduction in preoperative PSA, a change in the response to the PSMA-PET scan, and histopathological evidence of effect in most of the patients treated [35]. It is important to acknowledge that the reduction in preoperative PSA is not a relevant endpoint and does not impact the main survival outcomes. Neoadjuvant [177Lu]Lu-PSMA-617 has only been evaluated in phase I [36] or II trials [35], which makes it difficult to generalize the results. Further evidence is needed to support this promising treatment.

## 5. Intraoperative Approaches

Optimizing the cure does not always equal to intensifying the treatment. Indeed, reducing the aggressiveness of surgery using a targeted approach is mandatory to avoid unnecessary harm. Many preoperative and intraoperative strategies can be undertaken to maximize the radicality, minimizing complications. Research is now focused on trying to de-escalate the intraoperative treatment. In this direction, a recent analysis showed that unilateral nerve-sparing radical prostatectomy may be feasible and safe in patients staged with mpMRI in the absence of seminal vesicle invasion, with a small index lesion (<15 mm) and absence of homolateral clinically significant prostate cancer. Indeed, in these patients, the rates of EPE were around 5% [62,63]. Other previous studies also tried to use a nerve-sparing approach based on various radiological and pathological features to predict the risk of EPE [64]. The further stratification of HR patients will be the future management of localized PCa. Table 1 and Figure 1 present a synthesis of all the intraoperative strategies.

### 5.1. Lymphadenectomy for HR Prostate Cancer

LND in prostate cancer is now under debate; there are conflicting hypotheses on the effective role of this approach. Extended pelvic lymph node dissection (ePLND) is fundamental for optimal staging [48]. Recent findings showed that ePLND does not influence biochemical recurrence (BCR) but significantly increases clinical recurrence-free survival [65], particularly for patients with positive lymph nodes (pN1). This effect is not determined by a shorter time to salvage therapy but may be due to a tumor self-seeding mechanism [66]; thus, ePLND may be the possible explanation for the observed reduction in metastases [67]. Cancer cells might remain latent in the body for long periods before becoming active; removing all the metastatic nodes may lower the risk of early recurrences [42]. At the same time, it is not safe to perform a unilateral LND in HR PCa. Most of the patients with contralateral metastases have HR PCa, even though metastatic lymph nodes contralateral to the prostatic tumor location in the gland are overall a rare occurrence [40]. The exact extent of ePLND is not defined. When deciding the extent of the LND, it must be considered that the invasion of Cloquet’s ilioinguinal lymph node is extremely rare (1.2%) [39], so it may be spared during ePLND in patients without a sentinel lymph node in the Cloquet’s fossa or large anterior tumors [41,68]. For all these reasons, the combination of PSMA-PET with modern nomograms and the development of radio-guided and PSMA-guided surgery may be the future, presenting a potential opportunity to spare unnecessary perioperative morbidities for high-risk patients [69].

### 5.2. PSMA-Guided and Radioisotope-Guided Surgery

Gamma-emitting radionuclides, such as 99m-technetium, for radioactive tagging of PSMA ligands (e.g., 99mTc-PSMA-I&S) may be implemented for PSMA-based radio-guided surgery (PSMA-RGS). The literature on this topic is still limited, with conflicting results. The injection of 99mTc-PSMA-I&S before robot-assisted radical prostatectomy (RARP) and lymphadenectomy helps identify suspected lymph nodes. During the dissection of 297 lymph nodes, Yılmaz B. et al., found PSMA uptake in 18 lymph nodes, and the intraoperative probe counts correlated precisely with the pathological report. The sensitivity, specificity, accuracy, and negative and positive predictive values were all 100% [16]. However, a phase II prospective study demonstrated that PSMA-RGS accurately identified patients as pN1, and these patients exhibited nodal uptake also on preoperative 68Ga-PSMA-PET/MRI [37]. The intensification of diagnostic procedures including intraoperative techniques to better stratify patients with prostate cancer can lead to a reduction in surgical complications due to more invasive procedures that may be unnecessary. Lymph node metastases detection during surgery using a PSMA-targeted probe could serve as a guide for targeted treatment [16]. Although PSMA-RGS may aid surgeons in identifying LNI during ePLND, its sensitivity for detecting micrometastatic nodal dissemination is still insufficient [37]. Higher sensitivity and specificity were also found in the SENTINELLE study, where a 99mTC nanocolloid identified 141 out of 142 patients with node-positive disease after a sentinel node biopsy [38].

### 5.3. Indocyanine-Guided Lymphadenectomy

Another technique that may enhance diagnostic accuracy and consequently minimize complications is the use of indocyanine green (ICG) for guided rather than extended lymphadenectomy. In a recent randomized clinical trial, De Pablos-Rodriguez et al. utilized indocyanine green to identify prostatic lymphatic drainage, enabling them to perform a limited lymphadenectomy against extended pelvic lymphadenectomy (ePLND) during RP. The use of indocyanine for guided lymphadenectomy seems to ensure the same oncological outcomes with fewer complications, reducing the number of lymph nodes dissected [43]. Anyway, for both the ICG and PSMA-RGS, it is not clear whether the pathological staging could be affected by a reduction in the number of lymph nodes removed.

### 5.4. Intraoperative Artificial Intelligence Models to Guide Surgeons during Robotic Prostatectomy

Artificial intelligence is increasingly used in clinical practice. A preliminary study by Bianchi et al. evaluated the efficacy of augmented reality three-dimensional models in guiding intraoperative frozen section analysis during nerve-sparing RARP. Compared to traditional assessment based on mpMRI, the use of augmented reality models may reduce positive surgical margins (PSMs), specifically at the index lesion, potentially improving surgical outcomes in prostate cancer patients [44]. Another study with a broader patient cohort explored the role of AI models used during RARP [45]. Robotic surgery guided by 3D virtual models should be further investigated to understand the real impact in terms of PSM, since the results available are promising.

Moreover, Kwong et al. utilized artificial intelligence to assess extra-prostatic extension (EPE), enabling safer nerve-sparing prostatectomy. They developed, externally validated, and conducted an algorithmic audit of an AI-based tool called SEPERA (Side-specific Extra-Prostatic Extension Risk Assessment). In patients with pathologic EPE, SEPERA correctly predicted EPE in 68% of the 106 cases compared to the other models [47].

On this direction, Musi et al. recently proposed a phase III RCT to investigate augmented reality RARP compared to standard RARP, aiming to assess its efficacy in reducing positive surgical margins and improving secondary outcomes such as nerve-sparing approaches and erectile function recovery [46].

On the other hand, SEPERA demonstrated a superior net benefit compared to the other models in predicting EPE, allowing more patients to safely undergo nerve-sparing procedures [47]. Nevertheless, all the studies discussed still rely on manual models rather than automatized AI models.

Artificial intelligence and machine learning can provide better pathology insights, facilitating targeted patient care and potentially reducing overtreatment [44]. Nonetheless, further research with larger cohorts and longer follow-up periods is needed to validate these findings and assess long-term clinical outcomes.

## 6. Postoperative Treatments

Surgical treatment for HR non-metastatic PCa is sometimes followed by adjuvant radiotherapy with or without systemic treatment. Anyway, the scientific literature supports the use of early salvage radiotherapy (ESRT, administered when the postoperative PSA rises up to 0.5) rather than immediate adjuvant radiotherapy (IART, within 4 weeks–6 months after RP) for most of the patients with HR disease [48]. Some physicians base their choice on several risk factors, also considering the preference and quality of life of the patient. Table 1 and Figure 1 present the main findings related to postoperative treatment.

### 6.1. Adjuvant Therapies

There are several pathological features that can increase the risk of disease recurrence or progression: EPE, seminal vesicle invasion (SVI), extraprostatic organ invasion, positive surgical margins (R+), presence of positive lymph nodes at final pathology (pN1), and a higher Gleason score (GS). For patients with many risk factors and in particular those in which pN1 disease adjuvant treatments may potentially have a role, more high-quality studies are warranted to understand how to manage these patients with a higher risk of recurrence. Radiotherapy (RT) increases the rates of BCR-free survival, local control, and disease-free survival [48].

### 6.2. Role of Adjuvant Radiotherapy in pN1 Disease

According to EAU guidelines, pN1 at RP is classified as locally advanced disease. pN1 patients present a higher risk of recurrence, and for this reason, these patients might be potential candidates to receive adjuvant treatments. The choice of surveillance or treatment is generally based on tumor characteristics and the extent of nodal involvement [48]. Frohener et al. led a study on 495 pN1 patients treated with IART. During a median follow-up of 5.4 years, IART was independently associated with lower mortality in patients with HR features. In these pN1 patients, supplementary adjuvant androgen deprivation (<3 months) appeared to be superior to delayed treatment with a general lower mortality [70]. A multicenter study involved men with pT2-4 N0 or N1M0 disease. In the N1 subgroup, IART versus ESRT caused a reduction in all-cause mortality [71]. Patients with pN1 disease should be carefully managed since adjuvant therapy might be the best option to improve both overall and cancer-specific survival. However, the evidence available is too low to introduce its routine use in practice. Unfortunately, previous clinical trials were not focused on this specific subset of patients.

### 6.3. Androgen Deprivation Therapy as an Adjuvant Strategy

Studies supporting hormonal therapy alone as a postoperative strategy are limited, with few clinical trials that cannot be used as a reference for everyday clinical practice [51,52,54,72]. The European guidelines support the use of androgen deprivation therapies alone after RP only in pN1 patients, and the strength of the recommendation is weak [48].

Among the most recent studies, Ye et al. enrolled 189 HR or locally advanced prostate cancer patients who underwent a radical prostatectomy; they failed to demonstrate a difference in terms of decreasing 2-year PSA recurrence between patients who received complete androgen receptor blockade (CAB), LHRH agonist, or antiandrogen as an adjuvant treatment [52]. The investigators of the NCT02903368 tried to randomize patients to receive adjuvant apalutamide, abiraterone, prednisone, plus leuprolide for 12 months or an observation after an initial 6-month course of neoadjuvant therapy before surgery. The comparison of the rate of biochemical progression-free survival at 3 years post-RP was not possible due to the high drop-out rate [73].

Currently, some studies evaluating the role of ADT as an isolated adjuvant therapy are ongoing. The NCT01753297 trial evaluates the adjuvant effect of 9 months of triptorelin after RP in HR patients [54]. The NCT04523207 study evaluates apalutamide and ADT in HR non-metastatic patients who underwent RP [72]. The NCT05169112 aims to understand the role of a 12-month course of leuprolide after surgery [74]. The results of these studies could represent an important resource to improve the postoperative treatment of HR patients.

While the use of ADT alone after surgery is still controversial, ADT along with radiotherapy represents a cornerstone, especially in the management of HR patients. A recent phase III RCT involved 1716 patients who underwent ESRT after RP. Patients were randomized in three arms: (1) RT to the prostatic bed (PBRT), (2) PBRT and ADT for 6 months, (3) PBRT, RT to lymph nodes (PLRNT), and ADT. There was an apparent clinical benefit of adding ADT to RT, and for the first time, an increase in progression-free survival was seen also in patients undergoing PLRNT combined with ADT [51].

Both RADICAL-RT and GETUG-16 studies suggested that men who received ESRT should receive hormone therapy. Although the results showed an improvement of up to 6 months in disease-free-survival, this seems equivalent on both treatment strategies (ESRT vs. IART). Moreover, short-term versus long-term ADT should be tailored and based on the patients’ adverse features [49,50]. All these findings are consistent with a previous RCT which demonstrated the superiority of complete androgen blockade administered for 24 months combined with ESRT versus placebo in terms of overall survival. Most of the patients involved in this study had one or more HR features [75]. Finally, patients with at least one HR characteristic were treated with the addition of enzalutamide to the standard ADT along with ESRT in a recent clinical trial. Their initial results showed an apparent increase in terms of PFS without the increment of toxic-related events when adding enzalutamide in an early salvage setting; updates of this clinical trial may change the clinical practice in the future in terms of adjuvant therapy for HR PCa patients [53].

### 6.4. Chemotherapy

Only a few studies in the last 5 years investigated the use of chemotherapy as an adjuvant treatment after radical prostatectomy. Docetaxel has been the most studied in this setting. A phase III RCT investigated the role of docetaxel as an adjuvant treatment after RP in HR localized PCa without concurrent ADT. The trial randomized 298 patients to chemotherapy (docetaxel plus prednisone every 3 weeks for six cycles) or surveillance. They found no significant improvement in PFS, although potential benefits were found in a certain subset of patients (GS ≤ 7 and stage ≥ pT3b) [55]. A recent prospective study investigated the role of docetaxel in combination with ADT in a group of HR, locally advanced, non-metastatic PCa; however, the absence of a comparison group and the number of patients involved make it difficult to appreciate the real effect of the treatment on main survival outcomes [56]. The implementation of an adjuvant chemotherapy strategy is not a viable alternative to ADT since the evidence available did not demonstrate a superiority of chemotherapy over hormone therapy. Side effects can be another limit in the introduction of a treatment with uncertain benefits.

## 7. Conclusions

The treatment of HR PCa remains a challenge. New treatment strategies are emerging and gaining interest. The main purpose is to use the knowledge available to understand when it is appropriate to intensify the treatment or when it is required to intensify the preoperative staging to provide the best multidisciplinary treatment possible, based on the clinical, biochemical, molecular, and pathological features.

## Figures and Tables

**Table 1:** Summary of main findings.

	Treatment	Key Findings	Current Limitations
**Neoadjuvant treatment before radical prostatectomy**	**Androgen Deprivation Therapy**	NADT is not recommended by guidelines. It may reduce positive margin rate, disease volume, and positive lymph node rate without a substantial survival benefit [20].	Incomplete androgen suppression. No benefit in overall/disease-free survival. The most recent evidence is retrospective [21,22]. New clinical trials are more focused on combination therapies.
	**Abiraterone Acetate**	Enhances pathological response rates compared to ADT alone. Potential benefits in terms of BCR-free survival [23,24].	Mostly phase II trials. Limited experience in very high-risk patients [23,24].
	**Androgen Receptor Signaling Inhibitors**	ARSIs combined with ADT improves pathological response rates (e.g., MRD*, PR) and may have a longer BCR-free survival without an increase in perioperative complications and overall toxicity as compared to ADT [25,26,27,28].	No phase III trials. Surrogate outcomes are not strongly associated with main survival outcomes [29,30]. Limited experience in unresectable neoplasms [31].
	**Chemotherapy**	Docetaxel is the most studied and generally used in a multimodal setting. Improved metastasis-free, overall, and event-free survival but no difference in BPFS [32,33,34].	Overall modest impact in terms of overall survival. Potential grade 3–4 adverse effects (e.g., neutropenia). Concomitant ADT and/or adjuvant RT may act as confounders. Studies are not comparable [32,33,34].
	**[177Lu]Lu-PSMA-617**	Significant reduction in preoperative PSA. Histopathological effects reported [35].	Only phase I/II prospective studies [35,36].
**Intraoperative approaches to improve surgery**	**PSMA-Based Radioguided Surgery**	High accuracy in detecting metastatic lymph nodes. It can reduce unnecessary invasive procedures [37,38].	Not capable of detecting micrometastases. Insufficient power to draw conclusions [37,38].
	**Extended Lymphadenectomy**	Gold standard for staging. Improves clinical recurrence-free survival in pN1 patients. The combination of PSMA-PET results and nomograms as well as intraoperative guided LND may change the current surgical technique [39,40,41,42].	The extension of the lymphadenectomy is not defined. Unilateral lymphadenectomy for high-risk homolateral tumors is not safe [39,40].
	**Indocyanine-Guided Lymphadenectomy**	Comparable oncological outcomes to ePLND but lower complications rates [43].	Unclear impact on pathological staging. Only preliminary studies [43].
	**AI-Based Techniques**	AR models can reduce positive surgical margin rate. AI can improve prediction of EPE [44,45,46].	Requires larger, longer-term studies for validation [44,45,46,47].
**Postoperative treatments**	**Postoperative Radiotherapy**	Clear benefit in terms of BCR-free and disease-free survival. Early salvage preferred over immediate adjuvant for better QoL [48,49,50]. In pN1, increases overall and CSS.	Strict FU is needed especially for higher-risk patients electing for early salvage RT. The optimal fractioning of the dose is unclear [48,49,50].
	**Androgen Deprivation as Adjuvant**	Combined ADT + RT improves PFS, but ADT alone is not indicated, except for extremely selected cases. Combination of ARSIs, ADT, and RT may increase PFS without increasing toxicity [48,51,52,53].	Only few clinical trials support hormonal therapy alone [54].
	**Postoperative Chemotherapy**	It is not an alternative to ADT, but potential benefits were found for some subsets (GS < 7 and stage > pT3b) [55,56].	Few studies published with no significant improvement in PFS. Increased toxicity compared to standard treatment [55,56].

(NADT = neoadjuvant androgen deprivation therapy; ADT = androgen deprivation therapy; BCR = biochemical recurrence; ARSI = androgen receptor signaling inhibitor; MRD = minimal residual disease; PR = pathological response; BPFS = biochemical progression-free survival; RT = radiotherapy; LND = lymph node dissection; ePLND = extended pelvic lymph node dissection; AR = artificial intelligence; EPE = extra prostatic extension; QoL = quality of life; CSS = cancer-specific survival; PFS = progression-free survival; GS = Gleason score). MRD* definitions used in the studies reviewed: residual cancer burden <0.25 mm^3^; <5% prostate volume involved by the tumor, residual tumor < 5 mm.

## Data Availability

No new data were created or analyzed in this study. Data sharing is not applicable to this article.
